# Stabilization of Sur8 via PKCα/δ degradation promotes transformation and migration of colorectal cancer cells

**DOI:** 10.18632/oncotarget.23313

**Published:** 2017-12-14

**Authors:** Kug Hwa Lee, Woo-Jeong Jeong, Pu-Hyeon Cha, Sang-Kyu Lee, Do Sik Min, Kang-Yell Choi

**Affiliations:** ^1^ Translational Research Center for Protein Function Control, Yonsei University, Seoul, South Korea; ^2^ Department of Biotechnology, College of Life Science and Biotechnology, Yonsei University, Seoul, South Korea; ^3^ Department of Molecular Biology, College of Natural Science, Pusan National University, Pusan, South Korea

**Keywords:** Sur8, fibroblast growth factor-2, protein kinase C α/δ, Ras signaling, colorectal cancer

## Abstract

Scaffold proteins of the mitogen activated protein kinase (MAPK) pathway recruit protein kinase cascades to confer context-specificity to cellular signaling. Varying concentrations of scaffold proteins determine different aspects of signaling outputs. However, regulatory mechanisms of scaffold proteins are poorly understood. Sur8, a scaffold protein in the Ras-MAPK pathway, is known to be involved in cell transformation and migration, and is increased in human colorectal cancer (CRC) patient tissue. Here we determine that regulation of Sur8 stability mediates transformation and migration of CRC cells. Fibroblast growth factor 2 (FGF2) is identified as an external regulator that stabilizes Sur8. Protein kinase C-alpha and -delta (PKCα/δ) are also identified as specific mediators of FGF2 regulation of Sur8 stability. PKCα/δ phosphorylate Sur8 at Thr-71 and Ser-297, respectively. This phosphorylation is essential for polyubiquitin-dependent degradation of Sur8. Sur8 mutations, which mimic phosphorylation by PKCα/δ and destabilized Sur8, suppress the FGF2-induced transformation and migration of CRC cells. The clinical relevance of Sur8 regulation by PKCα/δ is indicated by the inverse relationship between PKCα/δ and Sur8 expression in human CRC patient tissues. Overall, our findings demonstrate for the first time a regulatory mechanism of Sur8 stability involving cellular transformation and migration in CRC.

## INTRODUCTION

Scaffold proteins are key regulators of cellular signaling pathways, which coordinate protein kinase cascades for proper and efficient signal transduction [1–3]. When cells encounter internal or external stimuli, scaffold proteins dynamically translocate to specific cellular locations to serve as physical platforms for the integration of signaling components into a spatially proximate orientation [1, 4]. It is recognized that regulations of scaffold proteins, especially regulating their concentrations, are crucial for the modulation of highly complex signaling networks. Various cellular states can be induced depending on the precise expression of scaffold proteins [2, 5]. Therefore, scaffold proteins require tight regulation of their expression levels to control complex physiological events.

The MAPK pathway which includes extracellular signal-regulated kinase (ERK), Jun N-terminal kinase (JNK), and p38, plays important roles in controlling cellular physiologies [6]. The Ras-ERK pathway has been implicated in fundamental cellular processes including cell growth, survival, and migration [7, 8]. Scaffold proteins in the Ras-ERK pathway function to modulate signaling intensity and duration in order to produce different cellular behaviors.

Suppressor of Ras-8 (Sur8), a scaffold protein of the Ras-ERK pathway, has been shown to be involved in cell growth, transformation, and migration via interaction with Ras and Raf [9, 10]. Increased Sur8 levels positively regulate Ras-mediated cellular processes. However, the mechanisms controlling Sur8 cellular levels have not yet been revealed.

In this study, fibroblast growth factor 2 (FGF2), a marker of epithelial to mesenchymal transition (EMT) and metastases [11, 12], is identified as a factor responsible for stabilizing Sur8. We investigate subsequent effects of FGF2-mediated Sur8 stabilization on transformation and migration of CRC cells. Sur8 stabilization contributes specifically to transformation and migration, but not normal growth, of CRC cells, mediated by FGF2. Amino acid sequence analyses of Sur8 raise the possibility that protein kinase C (PKC) might be involved in Sur8 phosphorylation. PKC-alpha and PKC-delta (PKCα/δ) are identified as specific kinases which phosphorylate Sur8. The stabilization of Sur8 by FGF2 is accomplished through PKCα/δ protein reduction, and the pathological relevance to the reverse regulation of Sur8 and PKCα/δ by FGF2 is observed through the inverse relationship between Sur8 and PKCα/δ protein levels in CRC patient tissue. We further characterize PKCα/δ phosphorylation of Sur8 at Thr-71 and Ser-297 by generating mutants for these phosphorylation sites. Our results reveal that phosphorylation of Sur8 is essential for its degradation and subsequent suppression of cell transformation and migration. Overall, this study provides a novel mechanism for Sur8 stabilization by FGF2 signaling resulting in transformation and migration of CRC cells.

## RESULTS

### FGF2 stabilizes Sur8 via inhibition of polyubiquitination-dependent proteasomal degradation

**Figure 1:**
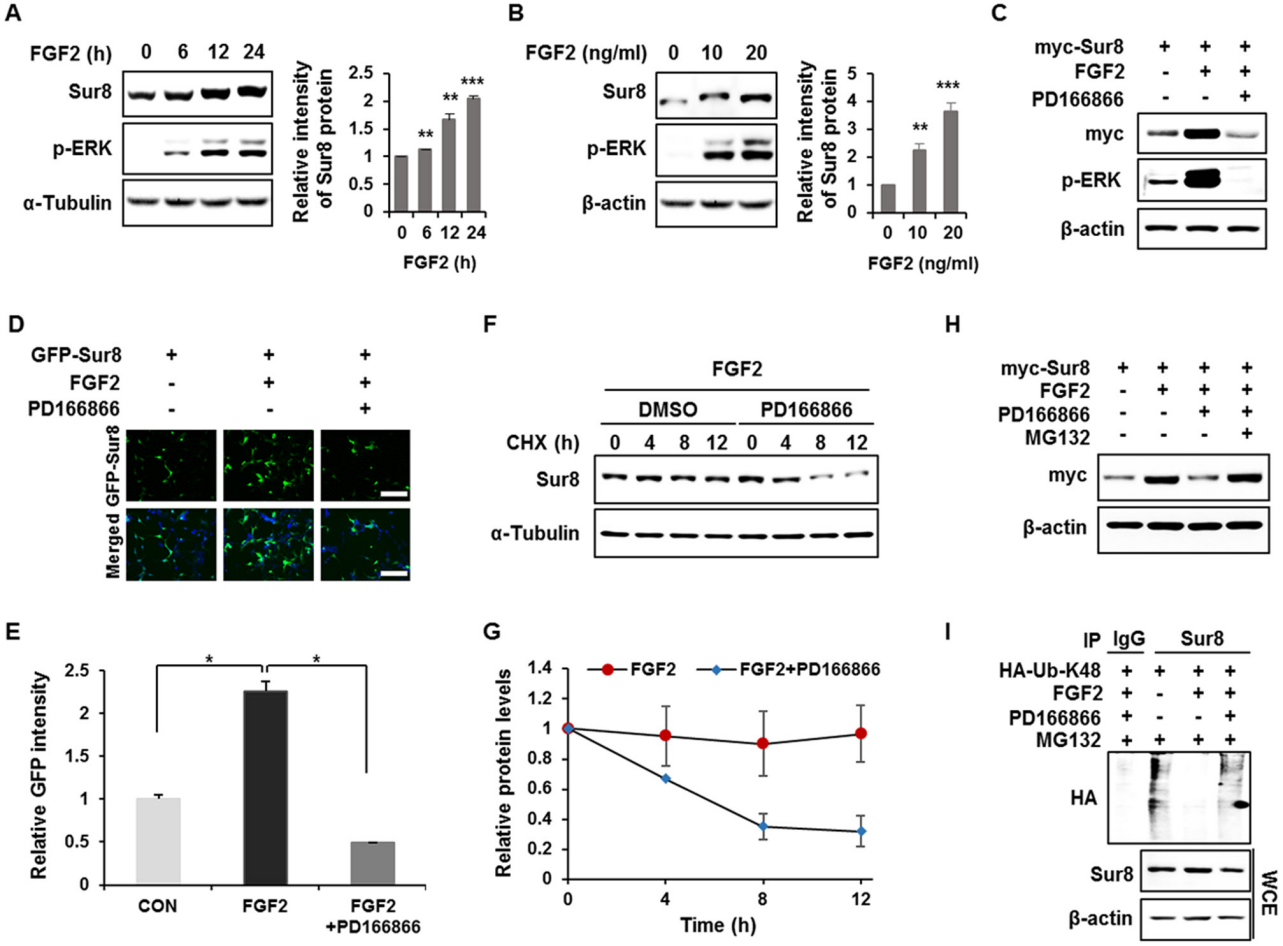
FGF2 stabilizes Sur8 via inhibition of polyubiquitination-dependent proteasomal degradation **(A, B)** HEK293 cells were treated with FGF2 in a time- (A) and dose- (B) dependent manner. **(C, D)** Cells were transfected with myc-tagged (C) or GFP-tagged (D) Sur8 plasmid. At 24 hours post-transfection, cells were treated with FGF2 (20 ng/mL) for 24 hours, followed by 2-hour pre-treatment with DMSO or a specific FGFR1 inhibitor, PD166866 (100 nM). Representative fields of GFP-positive cells (upper panel) and merged GFP and DAPI (lower panel) are shown (D). Scale bars, 500 μm. **(E)** Quantified data are shown in graph with mean ± SD from three independent experiments. ^*^P < 0.05. **(F, G)** Measurement of Sur8 turnover rate by a CHX chase assay. Cells were treated with 50 μg/mL CHX and with FGF2 alone or plus PD166866 for the indicated time periods. The Sur8 protein levels were examined by immunoblot (F) and quantitated (G). **(H, I)** Cells were transfected with myc-tagged Sur8 (H) or together with HA-tagged K48 ubiquitin (HA-Ub-K48) (I) and treated as indicated for 24 hours followed by 20 μM MG132 treatment for 4 hours before harvesting. Whole cell extracts (WCEs) were immunoprecipitated with an immunoglobulin G (IgG) control or Sur8 antibody (I). WCEs were subjected to immunoblot analysis using the indicated antibodies (A-C, F, H-I).

To identify a physiological signaling factor controlling Sur8, we tested the effects of several growth factors on Sur8 levels in human embryonic kidney 293 (HEK293) cells. FGF2, and to a lesser degree epidermal growth factor (EGF), increased Sur8 protein levels without changing Sur8 mRNA levels (Supplementary Figure 1A). FGF2 was more effective in stabilizing Sur8 as confirmed by a time-course analysis (Supplementary Figure 1B). We thus selected FGF2 for further investigation of its regulatory mechanisms of Sur8 stabilization. With FGF2 treatment, Sur8 levels increased, together with ERK activation, in a time- and concentration-dependent manner (Figure 1A and 1B). The increase in Sur8 levels, as well as the ERK activation by FGF2, was abolished with the addition of PD166866, an FGF2 specific inhibitor, as shown by both immunoblot (Figure 1C) and immunocytochemical analyses (Figure 1D and 1E). The effect of FGF2 inhibition on Sur8 stability was further confirmed by using another FGFR inhibitor, AZD4547 (Supplementary Figure 2). By treatment with cycloheximide, Sur8 levels were maintained up to 12 hours in the presence of FGF2. However, co-treatment with PD166866 substantially reduced the half-life of Sur8 (Figure 1F and 1G). FGF2-mediated Sur8 stabilization was attributed to the inhibition of the proteasomal degradation of Sur8 via suppression of its K48-linked polyubiquitination, as demonstrated by effects of MG132 (Figure 1H) and a ubiquitination assay (Figure 1I).

### Regulation of Sur8 stability is involved in FGF2-induced transformation and migration of CRC cells

**Figure 2:**
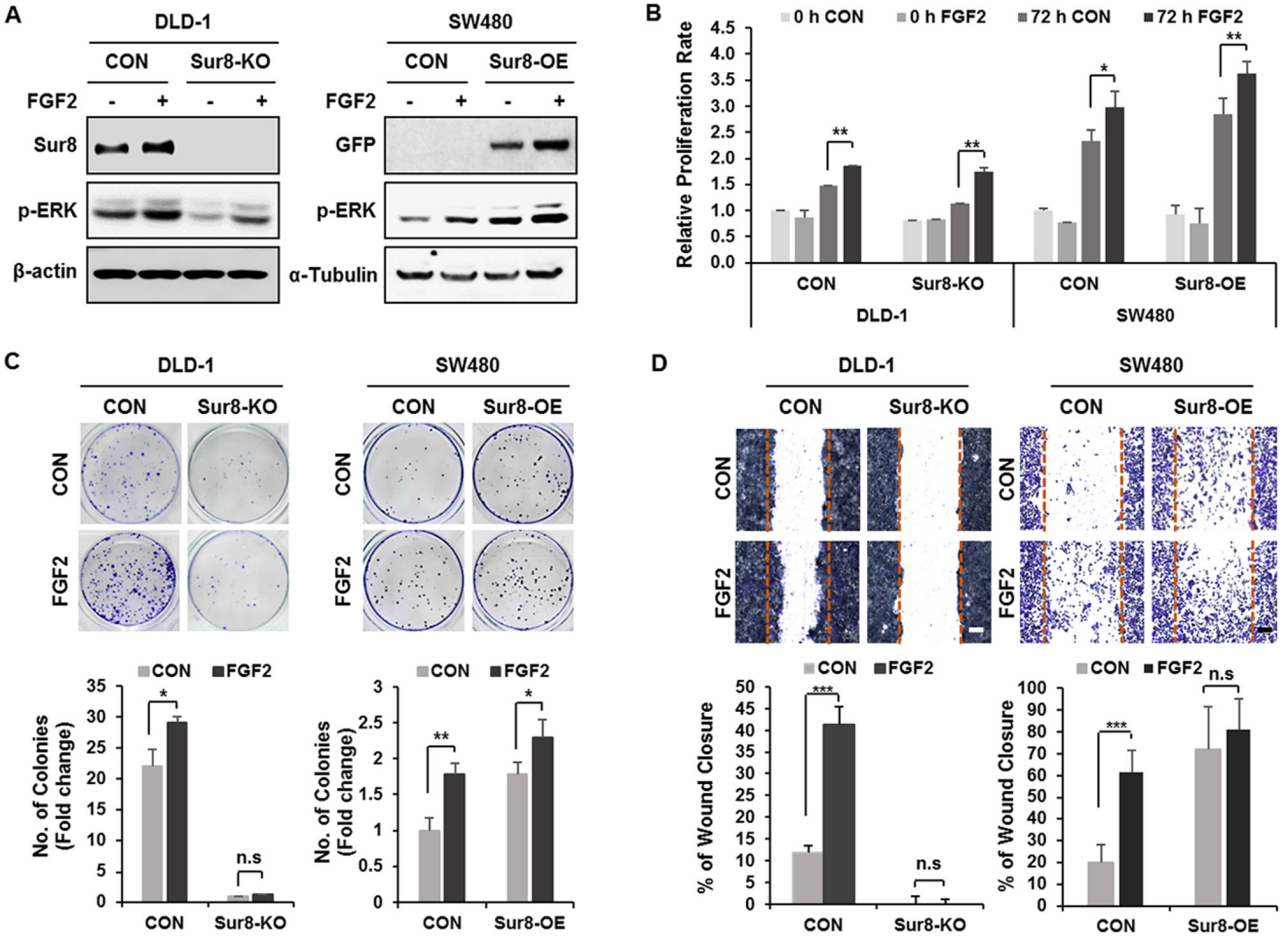
Effects of Sur8 level on FGF2-induced cellular transformation and migration **(A)** DLD-1 control (CON), Sur8-knockout (Sur8-KO) cells and SW480-CON, or stably expressing GFP-Sur8 (Sur8-OE) cells were treated with PBS (control) or FGF2 for 24 hours. WCEs were immunoblotted with the indicated antibodies. **(B-D)** After treating DLD-1 and SW480 cell lines as indicated, bioassays for measuring cell proliferation rate (B), foci-forming ability (C), and migration rate (D) were performed. All graphs represent mean ± SD of three independent experiments. ^*^P < 0.05, ^**^P < 0.01, ^***^P < 0.001. Scale bars, 500 μm.

We have previously observed overexpression of Sur8 in CRC patient tissues, as well as CRC cell lines, and identified a role of Sur8 in growth, transformation, and migration of CRC cells [13]. To investigate whether Sur8 stabilization by FGF2 is involved in neoplastic behavior of CRC cells, we first tested effects of FGF2 on Sur8 stability in DLD-1 human CRC cell line. As similarly observed in HEK293 cells (Supplementary Figure 1A), FGF2 was shown most effective one among the tested growth factors, and led to gradual increases in Sur8 stability, together with ERK activation in a time- and concentration-dependent manner (Supplementary Figure 3A-3C). We next examined whether Sur8 mediates FGF2-induced cellular responses by using DLD-1 and SW480 CRC cell lines having Sur8 knockout (KO) or overexpression (OE). ERK activities in Sur8-KO DLD-1 cells were substantially lower, compared with those in the corresponding parental DLD-1 cells, which express high levels of endogenous Sur8. The low ERK activity observed in Sur8-KO cells was only marginally restored by the addition of FGF2 (Figure 2A). However, basal ERK activity was significantly increased by Sur8-OE in SW480 cells, which express lower level of endogenous Sur8. The ERK activity was further increased by FGF2 treatment in Sur8-OE SW480 cells (Figure 2A). The observed increase in cell proliferation with FGF2 treatment in both Sur8-KO DLD-1 and Sur8-OE SW480 cells were not significantly different from that observed in parental cells (Figure 2B). However, FGF2-induced transforming potential was significantly reduced by Sur8-KO, and increased with Sur8-OE in DLD-1 and SW480 cells, respectively (Figure 2C). Similar effects of Sur8-KO and Sur8-OE were also observed in migration assays (Figure 2D). Overall, Sur8 stabilization plays a major role in FGF2-induced transformation and migration of CRC cells, without affecting the normal growth.

### PKCα and PKCδ mediate the FGF2-induced stabilization of Sur8

**Figure 3:**
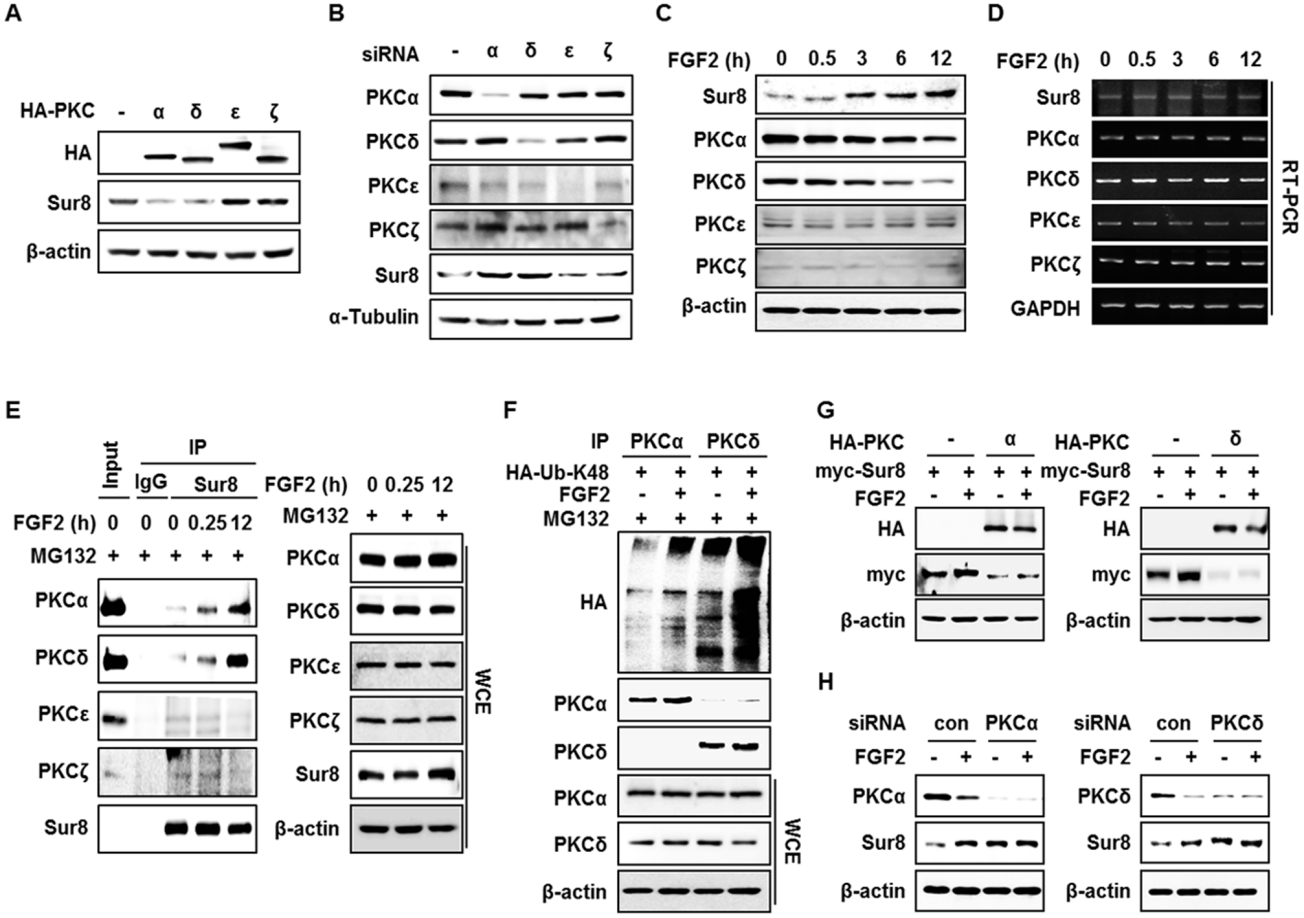
FGF2 stabilizes Sur8 through destabilization of PKCα and PKCδ **(A, B)** HEK293 cells were transfected with 0.5 μg of HA-tagged PKCα, δ, ε, or ζ in (A) and with 20 nMsiRNAs against PKCα, δ, ε, or ζ in (B). **(C, D)** Time-course stimulation of FGF2. Protein levels (C) and mRNA levels (D) of Sur8 and PKC isotypes were assessed by immunoblot and RT-PCR, respectively. **(E)** FGF2-dependent interaction of Sur8 with PKCα/δ. Cells were treated with FGF2 for the indicated time and MG132 for 4 hours before WCEs were collected for immunoprecipitation with either IgG control or anti-Sur8 antibody. **(F)** Cells were transfected withHA-Ub-K48 plasmid and treated with FGF2 for 12 hours and MG132 for 4 hours as indicated. WCEs were immunoprecipitated with anti-PKCα or PKCδ antibody. **(G, H)** Effects of PKCα/δ OE or KD on FGF2-mediated Sur8 stabilization. Cells were transfected with the indicated plasmids or siRNAs and treated with or without FGF2 for 24 hours at 24 hours post-transfection. WCEs were subjected to immunoblot analysis (A-C, E-H).

To further explore the underlying mechanisms of FGF2-induced Sur8 stabilization, we searched the database NetPhos 2.0 and found several putative phosphorylation sites on Sur8 (Supplementary Figure 4A). Moreover, PKC was suggested as the most potential kinase for the phosphorylation of Sur8 by NetPhos 3.1 sequence analysis (Supplementary Figure 4B). We tested if PKC regulates Sur8 stability by treatment with a pan-PKC inhibitor (Ro 31-8220), a classical PKC inhibitor (Gö6976), or a PKCδ specific inhibitor (rottlerin) in HEK293 cells. We observed an increase in Sur8 levels with inhibitor treatment (Supplementary Figure 5A-5C). Conversely, reduction of Sur8 levels was observed when PKC was activated by phorbol 12-myristate 13-acetate (PMA) (Supplementary Figure 5D). Furthermore, overexpression of PKCα and PKCδ, but not PKCε or PKCζ, reduced Sur8 levels (Figure 3A). The importance of PKCα and PKCδ (together denoted PKCα/δ) in Sur8 destabilization was further indicated by an increase of Sur8 levels after specific knockdown of these kinases (Figure 3B).

It is known that PKCs are activated by FGF2 [14]. Intriguingly, levels of PKCα/δ were specifically reduced during time-course treatment with FGF2 in HEK293 cells (Figure 3C). The reduction of PKCα/δ by FGF2 occurred at the protein level, as observed by the absence of change in mRNA levels of all PKC isotypes upon FGF2 treatment (Figure 3D). Consistently, the specific reduction of PKCα/δ by FGF2 correlated with FGF2-induced increase of PKCα/δ binding affinity to Sur8 after MG132 treatment (Figure 3E). This reduction can be attributed to polyubiquitin-dependent proteasomal degradation as shown by a ubiquitination assay (Figure 3F). Moreover, overexpression and knockdown of PKCα/δ abolished the effect of FGF2 on Sur8 stabilization (Figure 3G and 3H). In addition, even though both FGF2 and EGF were shown to increase Sur8 stability (Supplementary Figures 1A and 3A), overexpression of PKCα/δ was able to block FGF2-induced Sur8 stabilization only, indicating the specific roles of PKCα/δ in mediating FGF2 regulation of Sur8 (Supplementary Figure 6A and 6B).

### PKCα/δ promotes Sur8 degradation through phosphorylation of Thr-71 and Ser-297, respectively

**Figure 4:**
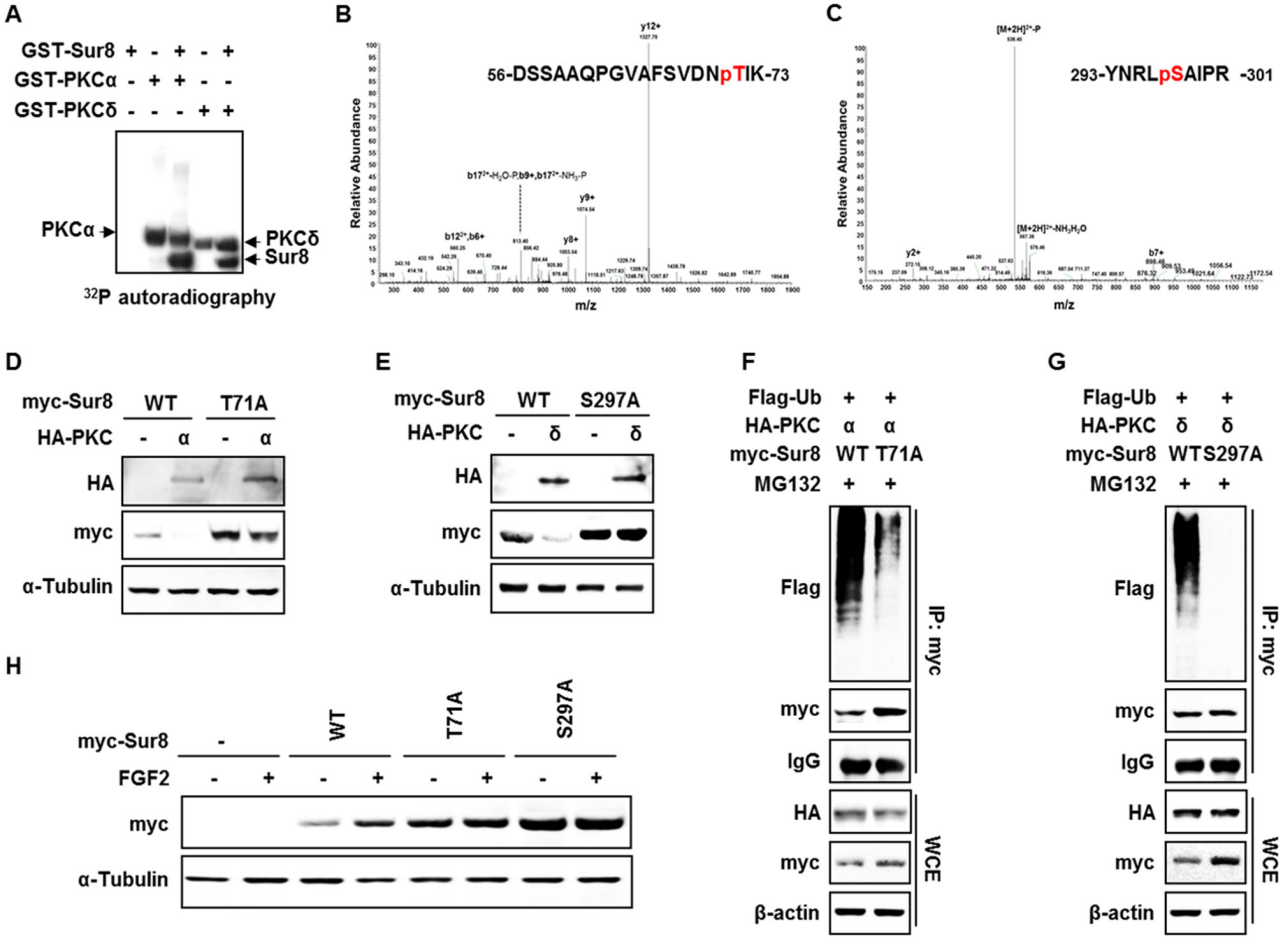
Phosphorylation of Sur8 at Thr71 and Ser297 by PKCα and PKCδ, respectively, are responsible for Sur8 degradation **(A)** Autoradiography of *in vitro* kinase assays with recombinant GST-Sur8, GST-PKCα, or GST-PKCδ alone, or GST-Sur8 plus GST-PKCα or GST-PKCδ. Autophosphorylated PKCα and PKCδ proteins, and phosphorylated Sur8 proteins are indicated. **(B, C)** Identification of phosphorylation sites of Sur8 by liquid chromatography tandem-mass spectrometry (LC-MS/MS). Graphs show representative mass spectrum of Sur8 depicting mass/charge (*m*/*z*) of identified PKCα (B) or PKCδ (C) phosphorylation sites. Spectra of the phosphopeptides in the digested Sur8 protein are shown. **(D, E)** Effects of nonphosphorylatable mutations of Sur8 on PKCα or PKCδ regulation of its stability. HEK293 cells were transfected with 1 μg of myc-Sur8-WT, myc-Sur8-T71A or myc-Sur8-S297A plasmid with or without HA-PKCα (D) or HA-PKCδ (E) plasmid. **(F, G)** Nonphosphorylatable mutation of T71 or S297 reduces Sur8 ubiquitination. Cells were transfected in the combinations of plasmids as indicated, followed by MG132 treatment for 4 hours before WCEs were immunoprecipitated with anti-myc antibody. **(H)** Cells were transfected with Sur8 nonphosphorylatable mutants and treated with FGF2 as indicated. WCEs were subjected to immunoblot analysis using the indicated antibodies (D-H).

To examine whether Sur8 is a direct substrate of PKCα/δ, we performed *in vitro* kinase assays using GST-fused PKCα and PKCδ (GST-PKCα and GST-PKCδ), and Sur8 recombinant protein. As shown by the assays, PKCα/δ phosphorylated Sur8, together with their auto-phosphorylation. (Figure 4A). Thr-71 and Ser-297 were identified as the PKCα/δ phosphorylation sites on Sur8 by LC/MS-MS analysis (Figure 4B and 4C). These phosphorylation sites are well conserved across species (Supplementary Figure 7). Moreover, comparison of the amino acid sequence of human Sur8 with that of other PKCα/δ substrates revealed that Sur8 possesses PKC consensus sequences similar to those of other PKCα/δ substrates (Supplementary Figure 8).

To further explore the regulation of Sur8 stability by phosphorylation, we utilized Sur8 nonphosphorylatable mutants, Sur8-T71A and Sur8-S297A, which cannot be phosphorylated by PKCα/δ, respectively. As shown by ablation of PKCα/δ-mediated degradation of the Sur8 nonphosphorylatable mutants, phosphorylation of Sur8 at Thr-71 and Ser-297 plays a critical role in its degradation (Figure 4D and 4E). Sur8-T71A, and to a greater extent Sur8-S297A, both substantially reduced the polyubiquitination of Sur8 by PKCα/δ (Figure 4F and 4G). Moreover, these phosphorylation sites are important for FGF2-induced Sur8 stabilization, as demonstrated by a comparably lower increase in levels of Sur8-T71A and -S297A after FGF2 treatment (Figure 4H). Together, Thr-71 and Ser-297 are essential residues for regulation of Sur8 stability by PKCα/δ and FGF2 signaling.

### Phosphorylation of Sur8 by PKCα/δ plays a critical role in transformation, migration, and invasion of CRC cells

**Figure 5:**
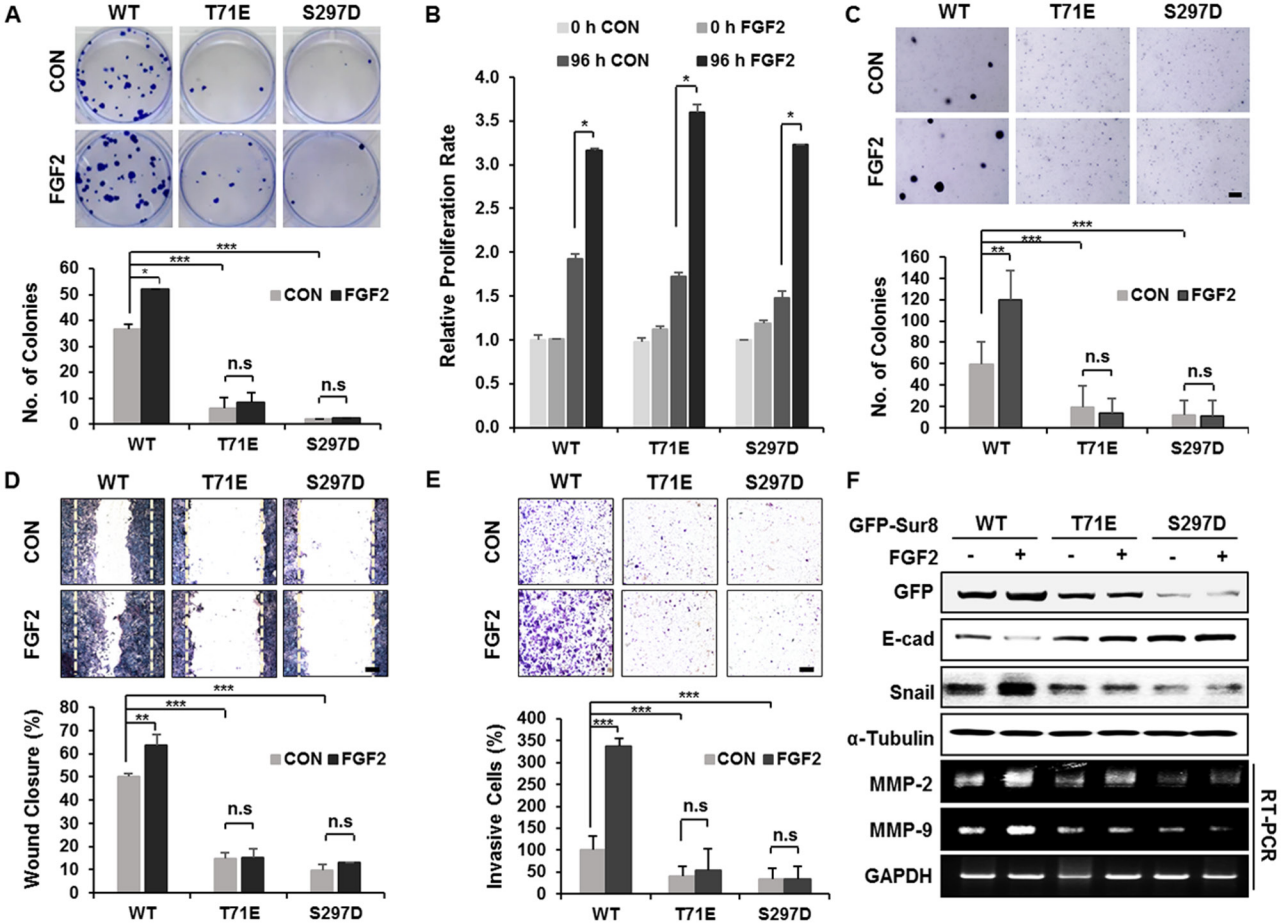
Wild-type Sur8, but not phosphomimetic mutations, mediates FGF2-induced cellular transformation, migration, and invasion **(A-F)** GFP-Sur8-WT, GFP-Sur8-T71E, or GFP-Sur8-S297D which were rescued in DLD-1 Sur8-KO cells were treated with PBS (control) or FGF2 and assessed for transformation, proliferation, migration, and invasion potential, and were subjected to immunoblot and RT-PCR analyses. Foci-forming assays were conducted for 2 weeks. Quantifications of colony number are shown in (A). Normal (B) and anchorage-independent (C) cell growth rates were determined by MTT and soft agar assays, respectively. Cells were scratched and allowed for wound closure for 48 hours after treatment with or without FGF2. Representative images of wound closure and quantification of relative wound closure efficiency are shown in (D). Invasion assays were performed using matrigel-coated chambers, and the relative quantification of invaded cells were measured as shown (E). Colonies and cells were stained with crystal violet (A, C-E). Statistical values were ^*^P < 0.05, ^**^P < 0.01, ^***^P < 0.001. Scale bars, 500 μm. WCEs were subjected to immunoblot analysis and total RNAs were subjected to RT-PCR analysis (F).

To characterize the importance of the PKCα/δ phosphorylation of Sur8 in the FGF2-mediated cellular processes of CRC cells, we generated cell lines with wild type Sur8 (DLD-Sur8-WT) or phosphomimetic mutants (DLD-Sur8-T71E and DLD-Sur8-S297D) rescued in DLD-1 Sur8-KO cell lines (DLD-Sur8-KO). As shown by immunoblot (Supplementary Figure 9A) and immunocytochemical (Supplementary Figure 9B and 9C) analyses, Sur8 stability was lower in DLD-Sur8-T71E and DLD-Sur8-S297D cells compared with DLD-Sur8-WT cells. Only marginal effects on Sur8 stability in DLD-Sur8-T71E and DLD-Sur8-S297D cells were observed with stimulation by FGF2 or inhibition of FGF2 by PD166866 (Supplementary Figure 9B and 9C). The focus-forming ability in DLD-Sur8-T71E and DLD-Sur8-S297D cells was also critically lower than in DLD-Sur8-WT cells (Figure 5A). Consistent with the no effect of Sur8-KO on FGF2-induced cell growth (Figure 2B), the growth rates in DLD-Sur8-T71E and DLD-Sur8-S297D cells were indistinguishable from that in DLD-Sur8-WT cells, regardless of FGF2 stimulation (Figure 5B). However, the effects of Sur8 on anchorage-independent cell growth, migration, and invasion rates were noteworthy. In DLD-Sur8-T71E and DLD-Sur8-297D cells, lower anchorage-independent cell growth, migration, and invasion were all observed, even with FGF2 stimulation (Figure 5C-5E). A concurrent increase in the protein levels of the epithelial cell maker E-cadherin, and reduction in the protein levels of the mesenchymal marker Snail and the mRNA levels of matrix metalloproteinase (MMP)-2 and MMP-9 were observed (Figure 5F).

We further evaluated the effect of blocking PKCα/δ phosphorylation of Sur8 by using SW480 cells expressing the nonphosphorylatable Sur8 mutations (SW-Sur8-T71A or SW-Sur8-S297A). Both transforming and migratory potential, though not normal growth, were higher in SW-Sur8-T71A and SW-Sur8-S297A cells compared to SW-Sur8-WT cells, without further effects of FGF2 (Supplementary Figure 10A-10C). Also, increased levels of Snail protein corresponded to the increased migratory potential of SW-Sur8-WT and mutant cells, as shown by immunoblot analysis (Supplementary Figure 10D). Overall, the PKCα/δ phosphorylation sites on Sur8 are critical for promoting Sur8-mediated and FGF2-regulated transformation, wound closure, and invasion.

### Low PKCα/δ expression correlates with elevated Sur8 levels in human CRC

**Figure 6:**
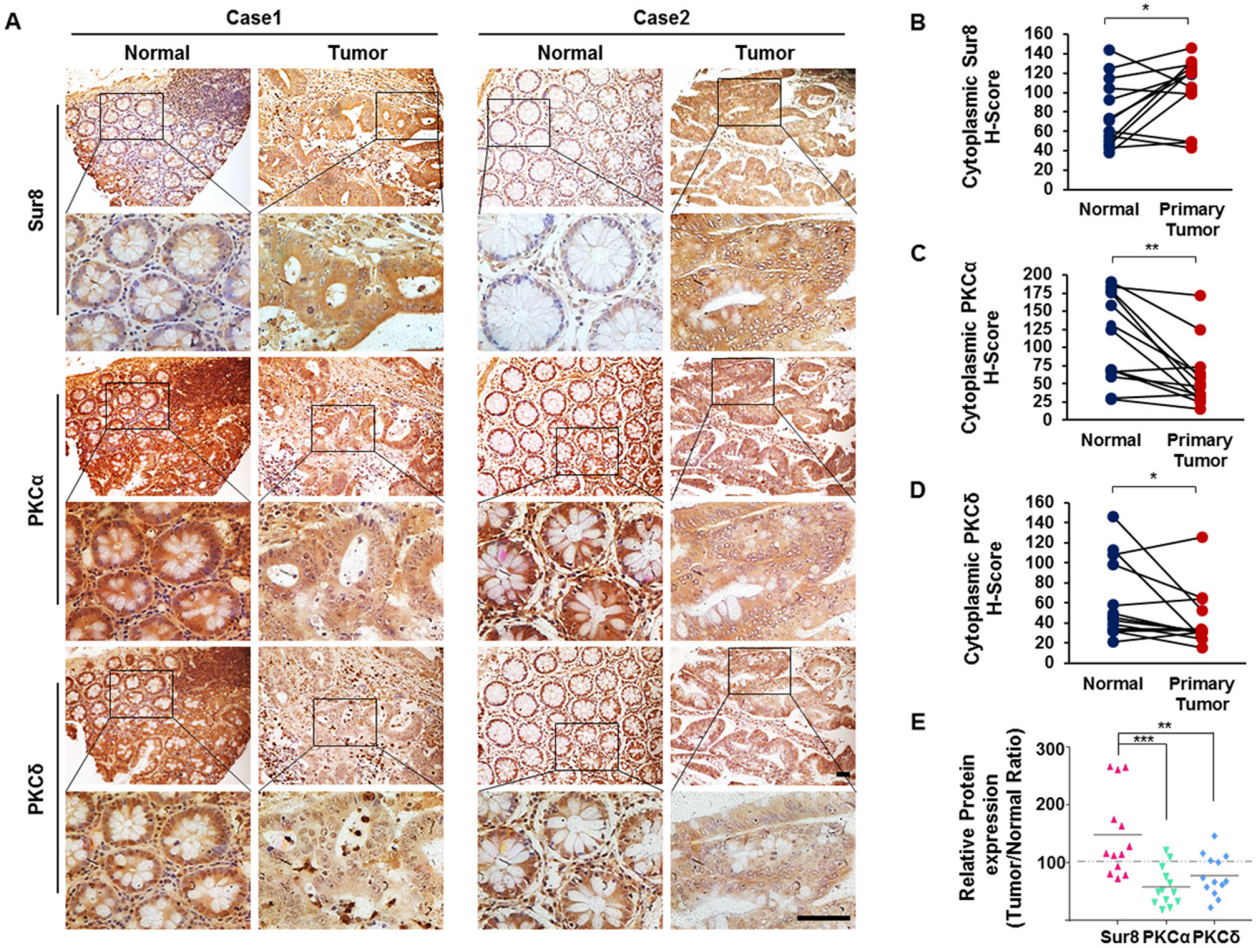
Expression of Sur8, PKCα, and PKCδ protein in human colorectal normal vs. tumor paired tissues **(A)** Normal (left panels) and tumor (right panels) colorectal tissues fixed on TMA slides were analyzed by DAB staining using Sur8 (top panels), PKCα (middle panels), and PKCδ (bottom panels) antibodies. Representative images are shown at 200× (upper) and 600× (lower) magnifications. Scale bars, 200 μm. **(B-D)** Quantitative analysis of Sur8 (B), PKCα (C), and PKCδ (D) was performed by comparing patient-matched adjacent normal (*n* = 13) colorectal tissue with tumor (*n* = 26) tissue based on H-Score. **(E)** Graphical image showing the inverse correlation between Sur8 and PKCα/δ expression of 13 sets of human colorectal adjacent normal and tumor tissues. All values were calculated using student’s *t*-test. ^*^P < 0.05, ^**^P < 0.01, ^***^P < 0.001.

PKCα/δ can exhibit different functions, either promoting or suppressing tumorigenesis in different cancer types [15]. In CRC, loss of PKCα or PKCδ promotes cell proliferation and transformation *in vitro* [16–18]. To investigate the clinical relevance of PKCα/δ regulation of Sur8 stabilization, we analyzed 13 sets of patient-matched tumor and adjacent normal tissue samples from human CRC. Immunohistochemical (IHC) analysis of these tissues revealed that Sur8 levels were higher in 9/13 (69.2%) CRC patient tumors compared with their adjacent normal tissue, whereas PKCα and PKCδ expression appeared lower in 11/13 (84.6%) and 8/13 (61.5%) of CRC tumors, respectively (Figure 6A-6D). Comparison of the protein expression ratios (tumor/normal) of Sur8, PKCα, and PKCδ from corresponding regions of patient-matched tumor and normal tissues further revealed significant negative correlations of Sur8 with both PKCα (*P*=0.0008) and PKCδ (*P*=0.0015) (Figure 6E). Thus, there are significant association between Sur8 and PKCα/δ levels in human CRC.

## DISCUSSION

Despite advances in the development of cancer therapeutics, current therapeutic strategies still require promising new targets capable of modulating key oncogenic proteins [19, 20]. Genetic alterations in *RAS* and *BRAF* are the driver mutations in cancer which is associated with resistance to current therapies and disease recurrence in various types of cancer, including CRC [21–23]. Emerging studies suggest that targeting the key molecular scaffolding complexes could be a novel therapeutic strategy to overcome current therapy limitations [24].

The scaffold proteins of the Ras/MAPK pathway, including IQGAP1, KSR1, and Sur8, have essential roles in growth, transformation, and migration of cancer cells. These scaffolding proteins are also more highly expressed in cancer cells than in normal cells. [13, 25–27]. Studies have demonstrated that disrupting the scaffolding functions of IQGAP1 protein deters acquired chemoresistance in Ras-driven tumors [24, 28], and inactivation of KSR by a small molecule inhibitor suppressed oncogenic Ras signaling [29]. Sur8 has been also shown to control tumor growth by affecting anchorage-independent cell growth, without affecting normal growth [10]. These studies suggest the possibility of scaffold proteins as potential ideal targets for cancer therapy.

Varying the expression levels of scaffold proteins is a key to control the kinetics and duration of Ras/MAPK signaling [30]. It is therefore important to understand the molecular mechanisms involved in regulation of these scaffold proteins, which could provide insight into developing novel cancer therapies in Ras-driven tumors. However, the regulatory mechanisms of scaffold proteins, and especially those which determine levels of scaffold proteins, are poorly understood.

**Figure 7:**
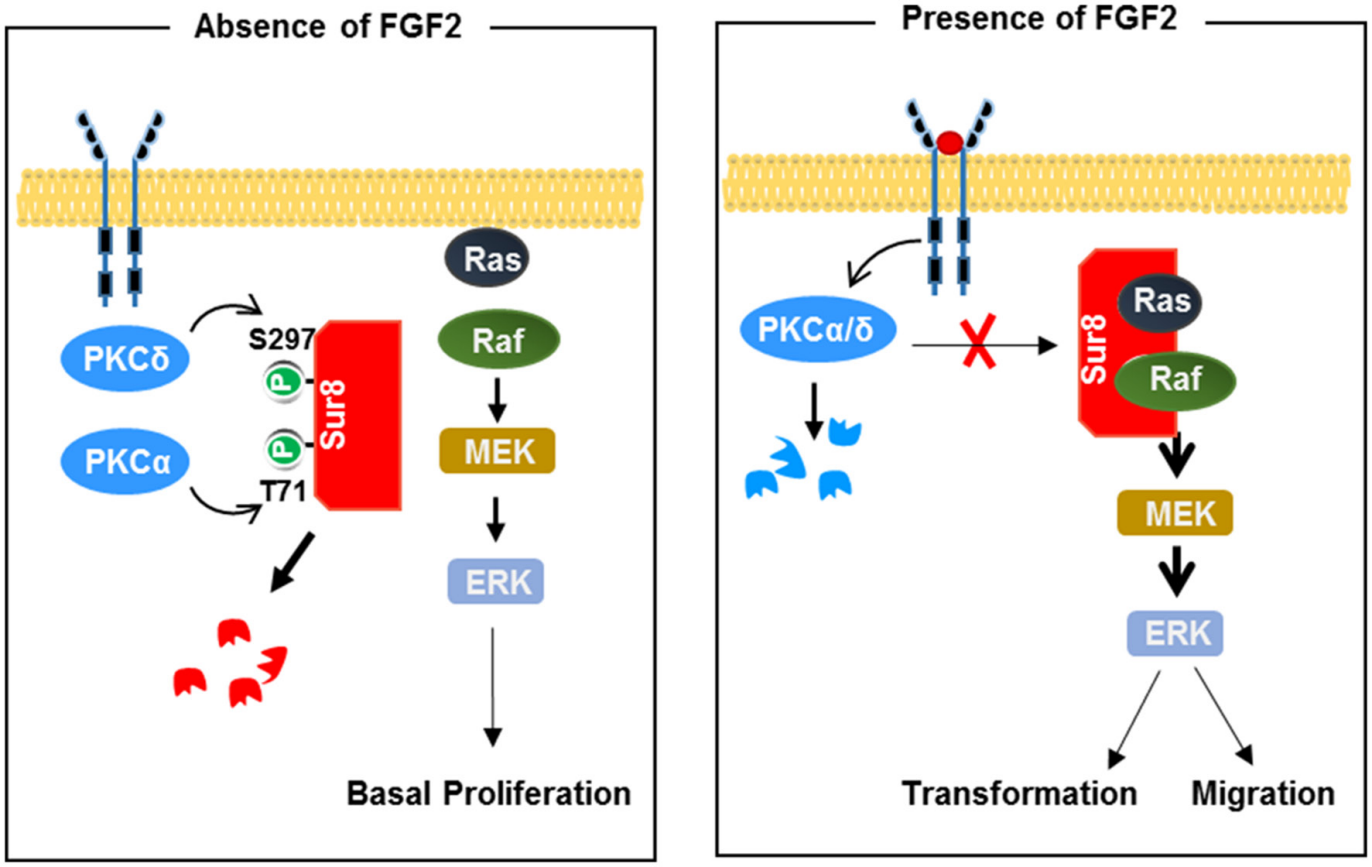
Model for Sur8 stability regulation by FGF2 signaling In the resting state (left panel), Sur8 is maintained at a low level by PKCα- and PKCδ-mediated phosphorylation at Thr-71 and Ser-297, respectively, and is subsequently degraded. This results in a basal level of cell proliferation. Upon FGF2 stimulation (right panel), Sur8 is maintained at a high level by stabilization through degradation of PKCα/δ. The stabilized Sur8 promotes transformation and migration of cells via activation of the Ras/ERK pathway.

We previously determined the functional roles of the Ras/Raf scaffold protein Sur8 in promoting CRC tumorigenesis and metastasis, and also melanoma-driven lung metastases via acceleration of Ras signaling activation [13, 25]. We have identified Sur8 as a potential molecular target for the suppression of oncogenic Ras-driven cancer. Here we provide a mechanistic understanding of Sur8 stabilization by FGF2 through blocking its phosphorylation by PKCα/δ (Figure 7).

FGF2 is known to play crucial roles in cell proliferation, transformation, and migration in CRC, though its functional mechanism is poorly understood [11, 31]. Our results show that FGF2 increases Sur8 expression through suppression of K48-linked polyubiquitination of Sur8, indicating that elevated FGF2 levels contributes to stabilizing Sur8. In addition, we show that PKCα/δ phosphorylate Sur8, reducing its stability. These results support a tumor suppressive role of PKCα/δ in CRC, consistent with recent findings [32]. The functional roles of PKCα/δ modulation of Sur8 were further delineated by the inverse regulation of Sur8 and PKCα/δ levels by FGF2, and the inverse expression levels of Sur8 and PKCα/δ in normal and tumor tissues of CRC patients. The reduced expression of PKCα/δ we observed in the tumor tissues of CRC patients is consistent with previous findings on reduced PKCα/δ expression in human CRC cell lines and murine intestinal tumors [17, 33]. Moreover, the role of FGF2 in PKCα/δ degradation correlates with a subsequent role of the PKC activator, TPA, in the degradation of PKCs [34]. There is a possibility that activation of FGFR by FGF2 induces an increase of intracellular Ca^2+^ levels and subsequently activates the known Ca^2+^-dependent PKC protease, calpain for the proteolysis of PKC [35–37].

Our mutational studies also demonstrate that Sur8 phosphorylation sites are key residues involved in the stabilization of Sur8. We showed that mimicking or blocking the PKCα/δ phosphorylation sites on Sur8 controls transformation and migration of CRC cells, including those mediated by FGF2. Notably, there was no effect on FGF2-meidated normal cell growth.

Collectively, our studies provide a novel mechanism for the regulation of Sur8 stability through pathologically relevant FGF2 signaling via degradation of PKCα/δ. The role of Sur8 in the specific regulation of transformation and migration of CRC cells, without affecting their normal growth, further highlights the importance of Sur8 as a potential target for cancer therapy, especially cancers with *Ras* mutations.

## MATERIALS AND METHODS

### Cell culture and use of reagents

HEK293, HEK293T, and human CRC cell lines (DLD-1 and SW480) were purchased from the American Type Culture Collection. The authentication of the cell lines were verified by short tandem repeat (STR) analysis provided by Cosmogenetech (Daejeon, Korea). Cells were propagated at 37°C and 5% CO_2_ in DMEM (Gibco, Grand Island, NY) or RPMI 1640 (Gibco) supplemented with 10% FBS (RMBI, Missoula, MA) and 1% penicillin-streptomycin (Gibco). The following reagents were administered: MG132 20 μM (Calbiochem, San Diego, CA); cycloheximide (CHX) and rottlerin 50 μg/mL and 6 μM, respectively (both from Santa Cruz, Santa Cruz, CA); EGF 20 ng/mL, FGF2 20 ng/mL, IGF1 50 ng/mL, and VEGF 20 ng/mL (all from Peprotech, Rocky Hill, NJ); Gö6976, Ro31-8220, and PD166866 at concentrations of 2.5 μM, 1 μM, and 100 nM, respectively (all from Sigma-Aldrich, St. Louis, MO); AZD4547 at a concentration of 100 nM (Selleckchem, Houston, TX); and phorbol-12-myristate-13-acetate (PMA) at a concentration of 100 nM (Cell Signaling Technologies, Beverly, MA).

### Transfection and siRNAs

Plasmid transfections were performed with Lipofector-EZ (AptaBio, Yongin, Korea) according to the manufacturer’s instruction. For transient knockdown of Sur8 and each PKC isozyme in HEK293 cells, siRNA duplexes of Sur8 and PKCs (Bioneer, Daejeon, Korea) were generated and conducted as previously described [25, 38].

### Cloning and site-directed mutagenesis

For generating GFP-tagged Sur8-overexpressing lentiviral plasmids, GFP was inserted into pLVX-IRES-Hyg vector (#632185, Clonetech, Palo Alto, CA) at *Not*I enzyme site prior to cloning Sur8. Human Sur8 complementary DNA (cDNA) was amplified by reverse transcription polymerase chain reaction (RT-PCR) using the following primers: forward 5′-ATAACCGGGATCCACCATGAGTAGTAGTTTAGGA-3′; reverse 5′-TCTAGAGGATCCGACCATGGCACGATATGG-3′. It was inserted into the pLVX-IRES-Hyg vector expressing GFP after digestion with *Bam*HI (Enzynomics, Daejeon, Korea).

For site-directed mutagenesis, point mutations of Sur8 were introduced by PCR using Pfu DNA polymerase (Invitrogen, Carlsbad, CA). The mutagenic oligonucleotides used for mutagenesis are shown in Supplementary Table 1. PCR reactions were run in the following condition: 15 cycles of 30 seconds at 95°C, then 1 minute at 54°C followed by 10 minutes at 68°C. The PCR products were digested with *Dpn*I (Enzynomics) for 1 hour for removal of the template plasmids. All mutant plasmids were verified by DNA sequencing (Cosmogenetech).

### Establishment of stable cell lines

To generate Sur8-knockout (KO) DLD-1 cells, DLD1 parental cells were transfected with human Sur8 CRISPR/Cas9 KO and HDR plasmids (Santa Cruz; sc-409478) according to the manufacturer’s instructions, and were selected with puromycin (Sigma-Aldrich) for 2 weeks.

To establish Sur8 rescue cell lines in Sur8-KO cells, HEK293T cells were transfected with pLVX-GFP-Sur8-WT, GFP-Sur8-T71E, or GFP-Sur8-S297D, together with the viral packaging psPAX2 and viral envelope pMD2G plasmids at 2:2:1 ratio, respectively, for viral production. Then, Sur8-KO DLD-1 cells were transduced with pLVX-GFP-Sur8-WT, GFP-Sur8-T71E, and GFP-Sur8-S297D lentivirus. To make SW480 cells lines stably expressing either Sur8-WT, Sur8-T71A or Sur8-S297A, parental SW480 cells were transduced with pLVX-GFP-Sur8-WT or the mutant lentiviruses. All transduced cell lines were selected with Hygromycin B (Duchefa, Haarlem, The Netherlands) for 2 weeks.

### RNA purification and RT-PCR

Total RNA was prepared and the mRNA levels of Sur8, PKCs, and GAPDH were measured as described previously [25, 38]. The mRNA levels of MMP-2 and MMP-9 were analyzed using the following primers (Bioneer): *MMP-2*, forward 5′-CTCAGATCCGTGGTGAGATCT-3′ and reverse 5′-CTTTGGTTCTCCAGCTTCAGG-3′; *MMP-9*, forward 5′-CAACATCACCTATTGGATCC-3′ and reverse 5′-CTGGGTGTAGAGT CTCTCGCT-3′.

### Immunocytochemistry, immunoprecipitation, and cellular ubiquitination assays

For immunocytochemistry assays, GFP-tagged Sur8-expressing cells grown on p-L-O and fibronectin-coated cover glasses (Sigma-Aldrich) were fixed with 4% paraformaldehyde (PFA) for 10 minutes. After washing with PBS, cells were counterstained with 4′, 6-diamidino-2-phenylindole (DAPI; Sigma-Aldrich) prior to mounting.

Immunoprecipitation was performed as previously described [25]. For ubiquitination assays, HEK293 cells transfected with plasmids expressing ubiquitin were lysed with lysis buffer (10 mM Tris-HCl pH 7.5, 150 mM NaCl, 1 mM EDTA, 0.1% NP-40, 10 mM NaF, 1 mM Na_3_VO_4_, 1 mM PMSF, and protease inhibitors) and then performed in a process similar to that of the immunoprecipitation assay.

### Purification of recombinant proteins and *in vitro* kinase assay

Glutathione-S-transferase-(GST)-tagged Sur8-WT was inserted into a pGEX-4T-1 vector using *Xho*I and *Bam*HI restriction sites and expressed in *Escherichia coli.* Cell lysates were incubated with glutathione-sepharose-4B bead (Sigma-Aldrich) for 1 hour at 4°C and GST-Sur8 recombinant proteins were eluted with reduced glutathione (Sigma-Aldrich). *In vitro* kinase assays were performed by incubation of purified GST-tagged Sur8 with either GST-PKCα (Signal Chem) or GST-PKCδ (Signal Chem) for 2 hours at 30°C in 20 μL of reaction buffer containing 50 mM Tris-HCl (pH 7.5), 10 mM MgCl_2_, 1 mM DTT, 10 mM ATP, and 5 μCi of [γ-^32^P] ATP. The reaction mixtures were resolved by SDS-PAGE, and phosphorylated Sur8 was visualized by autoradiography.

### Cell proliferation, soft agar, and colony-forming assays

For proliferation assays, cells were seeded at a density of 5 × 10^3^ cells/well in a 96-well culture plate and treated with or without FGF2 (20 ng/mL) for 72 hours. Cell growth was investigated by a 3-(4,5-dimethylthiazol-2-yl)-2,5-diphenyltetrazolium bromide (MTT)-based semi-automated colorimetric assay according to the manufacturer’s instructions.

Anchorage-independent soft agar and the foci formation assays were performed as previously described [13].

### Migration and invasion assays

For migration assays, cells were grown to confluence in a 12-well plate. Following 10 μg/mL mitomycin C treatment (Roche, Indianapolis, IN) for 1 hour to inhibit proliferation, cells were wounded using a 200 μL pipette tip and further cultured with or without FGF2 for 48 hours. The percent of wound closure was measured as the relative ratio of residual wound area and the original wound area.

For invasion assays, 3 × 10^4^ cells/ 200 μL were seeded on matrigel-coated invasion chambers (BD Bioscience, Bedford, MA) in a 24-well plate with or without FGF2. Cells were allowed to invade for 18 hours. The cells were fixed using 4% PFA stained with 0.1% crystal violet. Images were taken under microscope at 40× magnification.

### TMA and immunohistochemistry

The colorectal cancer TMA (NBP-47195) was purchased from Novus Biologicals. The TMA specimens consisting of 16 pairs of patient-matched adjacent normal and tumor tissues were subjected to immunohistochemistry analysis, following the procedure as previously described [25]. Images of each specimen were taken using a bright field microscope (Nikon ECLIPSE 80i). For the measurement of the cytosolic expression levels of proteins, the TMA slides were quantified using the IHC profiler plugin [39].

### Statistical analyses

Statistical analyses were performed using Microsoft Excel or GraphPad Prism. All data are represented as the mean ± standard deviation. Statistical differences were determined using Student’s *t*-test and considered statistically significant as follows: ^*^P < 0.05, ^**^P < 0.005, ^***^P < 0.001.

## SUPPLEMENTARY MATERIALS FIGURES AND TABLE


