# Supplementary material for: Stabilization of Sur8 via PKCα/δ degradation promotes transformation and migration of colorectal cancer cells
Source: Oncotarget. 2017 Dec 14;8(70):115596–608. doi: 10.18632/oncotarget.23313 (PMC5777796; doi:10.18632/oncotarget.23313)
Supplement: Supplementary file 1 [file oncotarget-08-115596-s001.pdf]

Stabilization of Sur8 via PKCα/δ degradation promotes transformation and migration of colorectal cancer cells

SUPPLEMENTARY MATERIALS

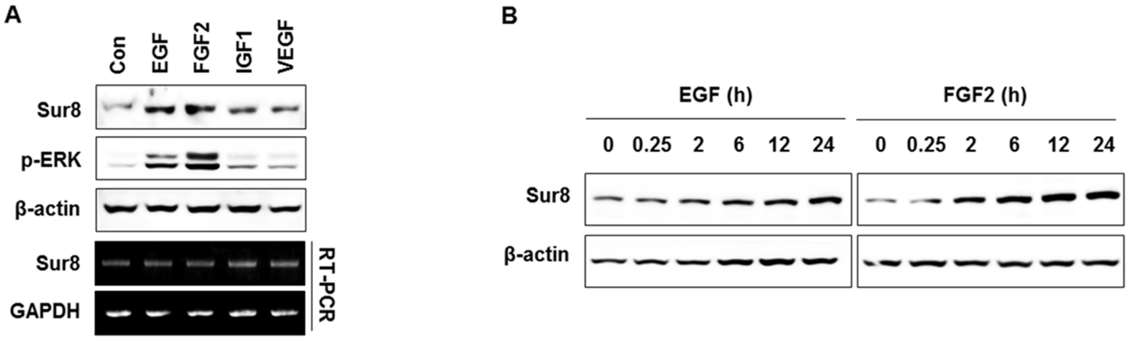

Supplementary Figure 1: Effects of growth factors on Sur8 protein levels. (A) HEK293 cells were treated with PBS (control); 20 ng/mL EGF, FGF2, or VEGF; or 50 ng/mL IGF-1 for 24 hours. WCEs were immunoblotted against Sur8, p-ERK, and β-actin, and mRNA levels of Sur8 and GAPDH were detected by using RT-PCR. **(B)** Cells were treated with 20 ng/mL of EGF and FGF2 at six different time points as indicated. WCEs were immunoblotted against Sur8 and β-actin.

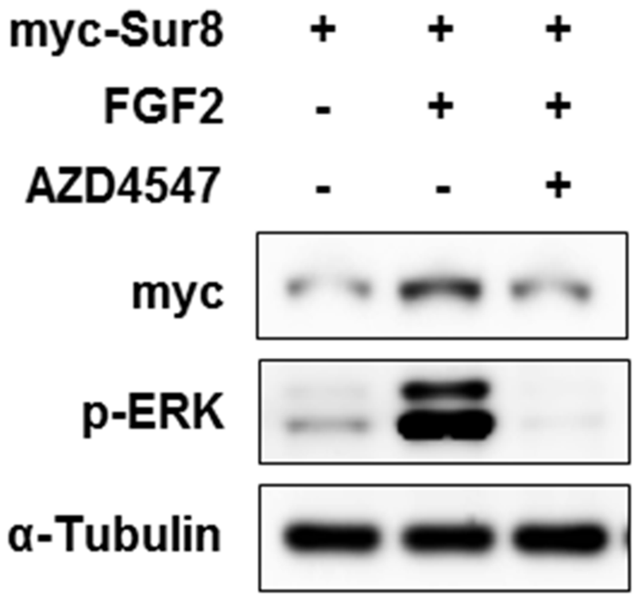

Supplementary Figure 2: Effects of AZD4547 on Sur8 stability. HEK293 cells were transfected with myc-tagged Sur8. At 24 hour post-transfection, the cells were treated with FGF2 alone or together with a selective FGFR inhibitor, 100 nM of AZD4547 for 24 hours. WCEs were subjected to immunoblotting with anti-myc, anti-p-ERK or anti-α-Tubulin antibody.

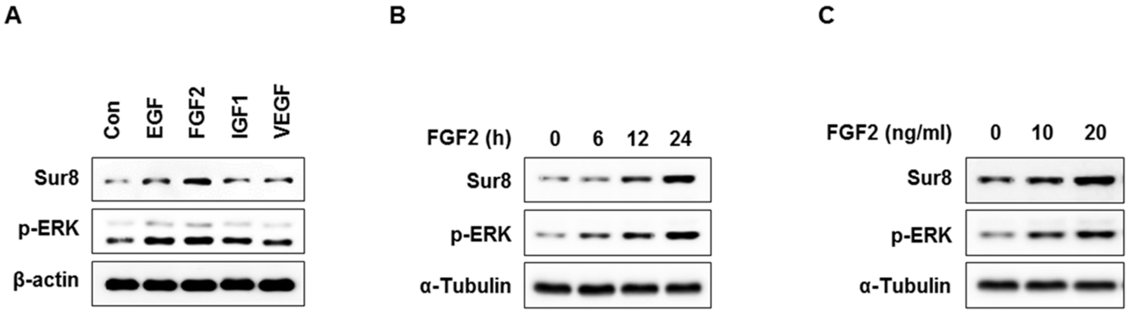

Supplementary Figure 3: Effects of growth factors on Sur8 stability in DLD-1 cells. **(A)** DLD-1 human CRC cellswere treated with PBS (control); 20 ng/mL EGF, FGF2, or VEGF; or 50 ng/mL IGF-1 for 24 hours. **(B, C)** DLD-1 cells were treatedwith FGF2 in a time- (B) and concentration- (C) dependent manner, as indicated. WCEs were immunoblotted against the indicated proteins (A-C).

A

Serine Prediction

Residue	Score
S11	0.996
S20	0.995
S31	0.942
S38	0.978
S57	0.801
S58	0.647
S67	0.794
S85	0.986
S86	0.934
S108	0.938
S118	0.986
S160	0.988
S164	0.985

Threonine Prediction

Residue	Score
T45	0.509
T71	0.865
T81	0.931
T205	0.813
T206	0.964
T402	0.521

Tyrosin Prediction

Residue	Score
Y523	0.939

B

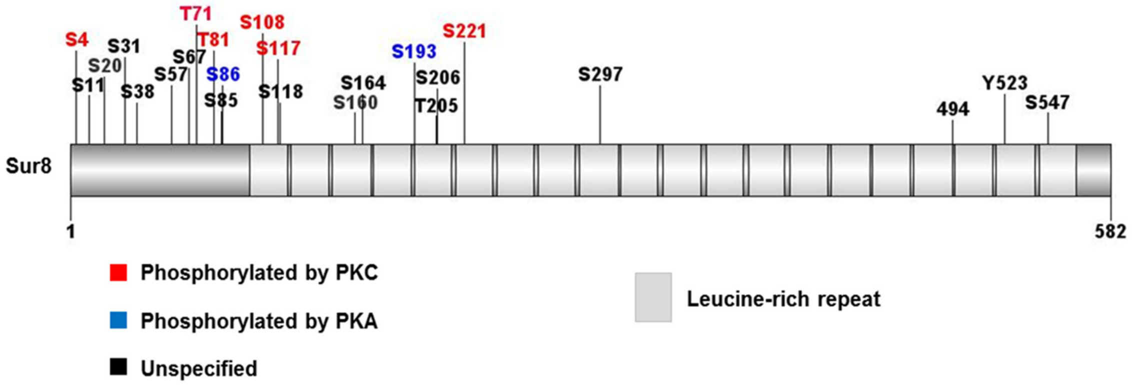

Supplementary Figure 4: Prediction of putative amino acid residues and kinases for phosphorylation in human Sur8. **(A)** Protein sequences of human Sur8 were analyzed by using NetPhos 2.0 (http://www.cbs.dtu.dk/services/NetPhos-2.0/). Possible phosphorylated residues having output scores above the threshold of 0.5 are shown. **(B)** Putative kinases and their predicted phosphorylation on amino acid residues of Sur8 were predicted using NetPhos 3.1 (http://www.cbs.dtu.dk/services/NetPhos/) with output scores above 0.75. Out of 24 possible phosphorylated residues of Sur8, 6 amino acid residues (red) were predicted for PKC phosphorylation sites, 2 amino acid residues (blue) for PKA phosphorylation, and the rest (black) were unspecified. Scores over 0.5 with a maximum score of 1 indicate that the residue is a possible phosphorylation site.

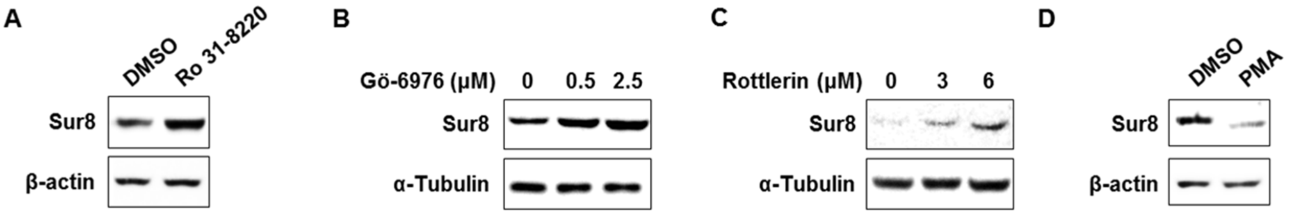

Supplementary Figure 5: Effects of chemical activation and inhibition of PKC on Sur8 protein level. (A-D) HEK293 cells were treated with a pan-PKC inhibitor, Ro 31-8220 (A); a classical PKC inhibitor, Gö6976 (B); a PKCδ inhibitor, rottlerin (C); and a pan-PKC activator, Phorbol 12-myristate 13-acetate (PMA) (D) for 24 hours. WCEs were immunoblotted for Sur8 and β-actin.

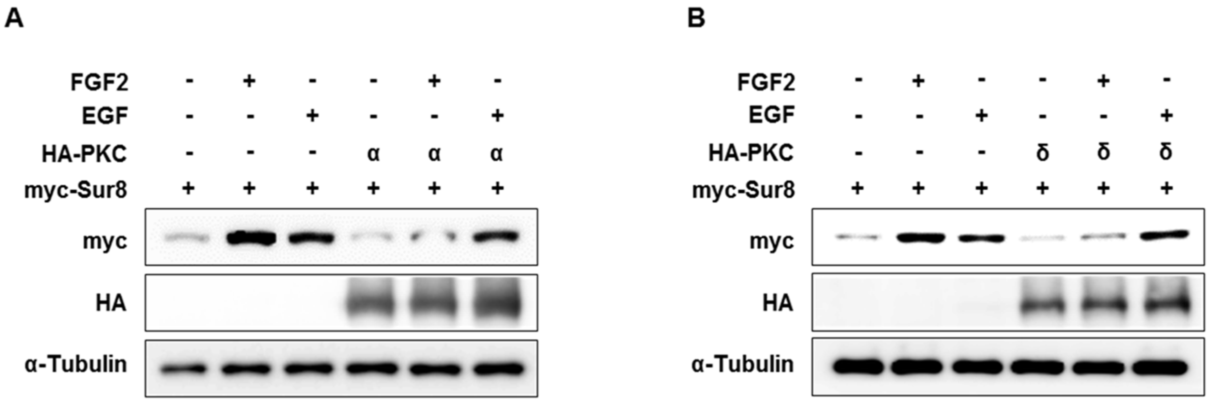

Supplementary Figure 6: Effects of PKCα/δ overexpression on FGF2- or EGF-induced Sur8 stabilization. (A, B) HEK293 cells were transfected with myc-tagged Sur8 and HA-tagged PKCα (A) or PKCδ (B). At 24 hour post-transfection, cells were treated with FGF2 or EGF for 24 hours as indicated. WCEs were subjected to immunoblotting against HA, myc, and α-Tubulin.

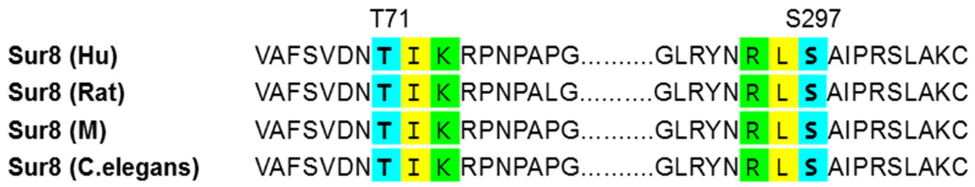

Supplementary Figure 7: The PKCα and PKCδ phosphorylation consensus sequences of Sur8. Alignment of Sur8 amino acid sequences from various species demonstrates high evolutionary sequence conservation at T71 and S297, the phosphorylation sites for PKCα and PKCδ, respectively. Phosphorylation sites are highlighted in blue and the remaining consensus sequences are highlighted as indicated.

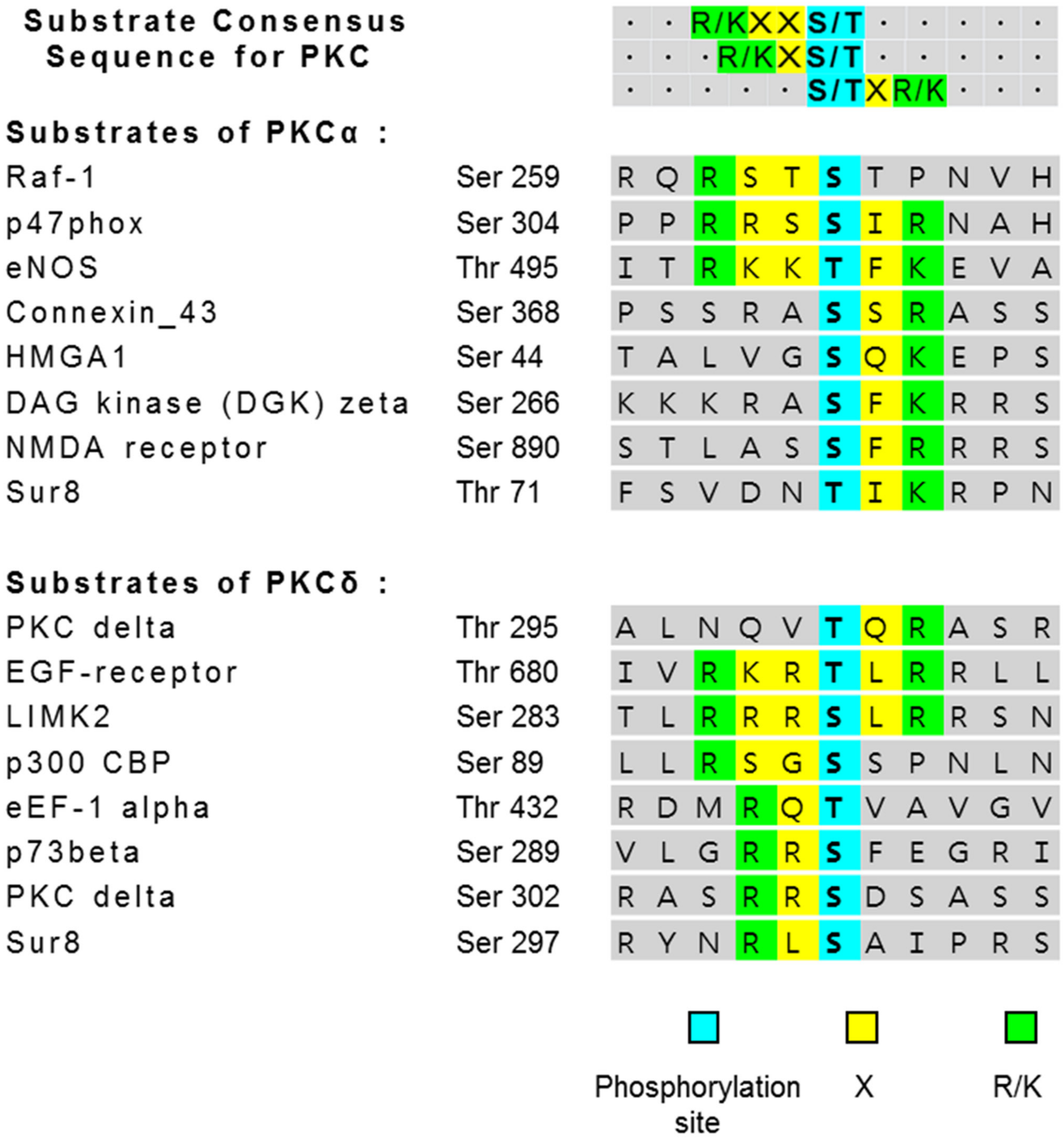

Supplementary Figure 8: Alignment of the amino acid sequences of Sur8 with those of other known PKCα and PKCδ substrates. Sequences of human Sur8 and other known substrates of PKCα and PKCδ are shown for comparison. The PKC consensus sequences [R/K]XX[pS/pT], [R/K]X[pS/pT], or [pS/pT]X[R/K] are indicated as phosphorylation sites shown in blue, the remaining consensus residues R/K in green, and any amino acid X in yellow.

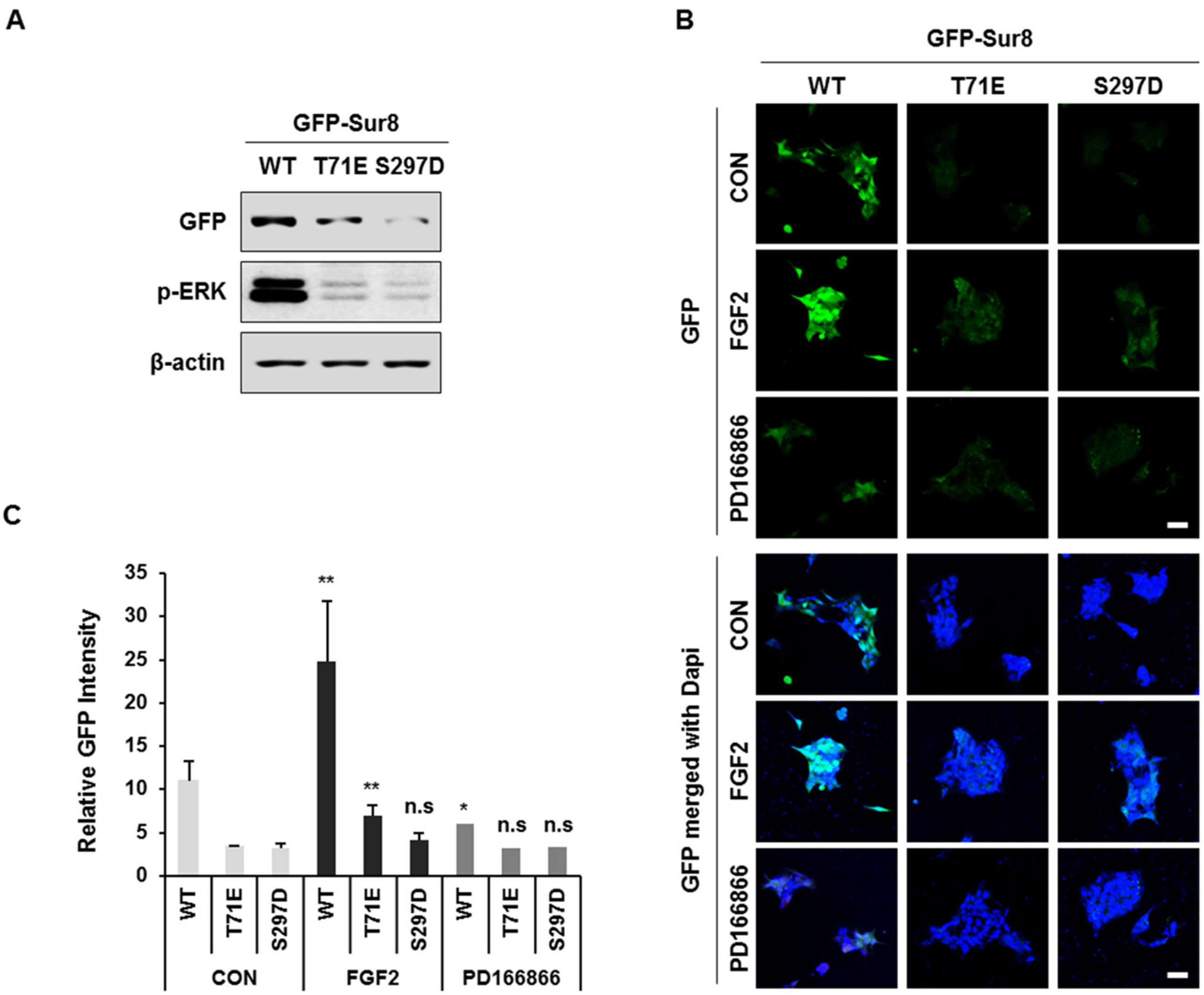

Supplementary Figure 9: Effects of Sur8 mutations mimicking PKCα/δ phosphorylation on Sur8 level and ERK activity. (A) WCEs from GFP-Sur8-WT, GFP-Sur8-T71E, or GFP-Sur8-S297D which were rescued in DLD-1 stable cell lines were immunoblotted with anti-GFP, anti-p-ERK or anti-β-actin antibody. **(B, C)** Fluorescent images for GFP in GFP-Sur8-WT, GFP-Sur8-T71E, or GFP-Sur8-S297D DLD-1 stable cell lines treated with PBS (control), FGF2, or PD166866 for 24 hours. Cell nuclei were stained with DAPI. Scale bars, 100 µm. $^{*}P < 0.05$, $^{**}P < 0.01$.

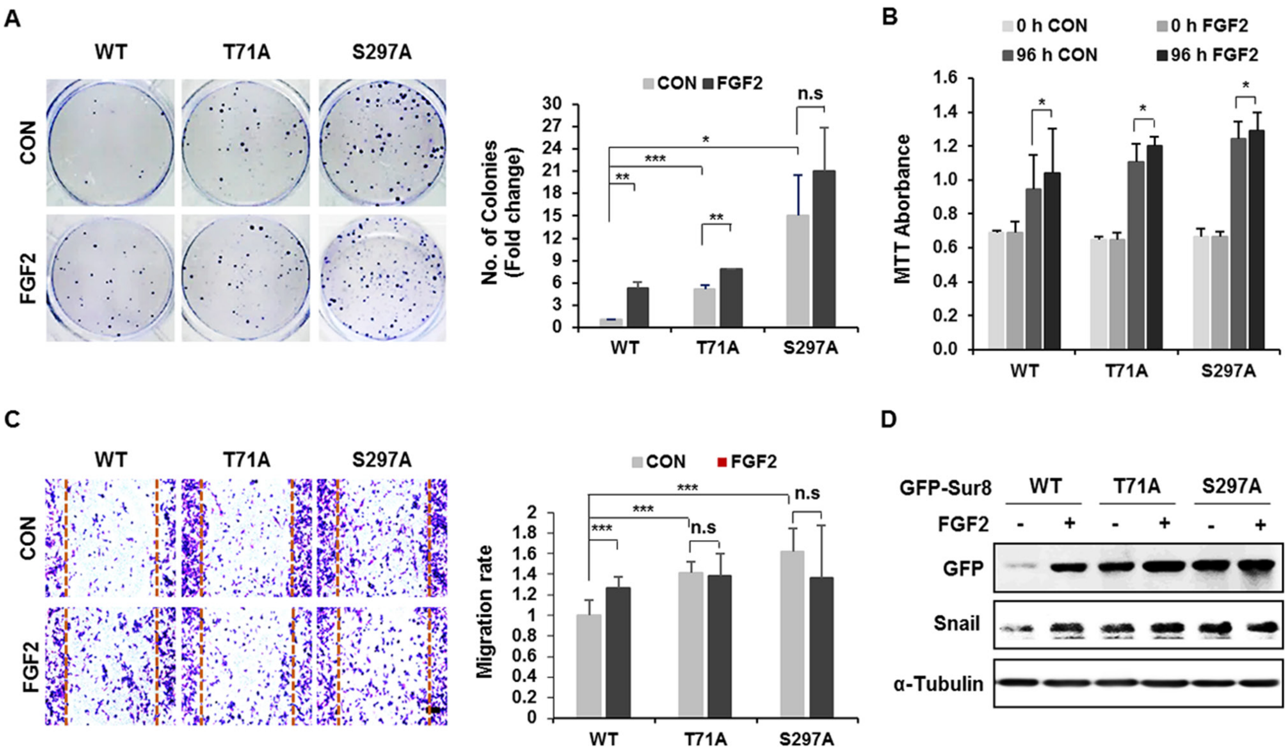

Supplementary Figure 10: Effects of nonphosphorylatable mutations of Sur8 on transformation, proliferation, and migration of SW480 cells. (A-D) SW480 cells expressing GFP-Sur8-WT, GFP-Sur8-T71A, or GFP-Sur8-S297A were treated with control or FGF2, and assessed for transformation, proliferation, and migration potential, and immunoblot analysis. Foci-forming assays were conducted for 2 weeks. Quantification of colony number is shown on right (A). Anchorage-dependent cell growth rates were determined by MTT assay (B). Number of migrated cells was counted after 36 hours of wound scratch to show migratory potential. Scale bars, 500 µm (C). Colonies and cells were stained with crystal violet (A, C). All the values are calculated by student's *t*-test. $^{*}P < 0.05$, $^{**}P < 0.01$, $^{***}P < 0.001$. WCEs were immunoblotted for detection of the EMT-related mesenchymal marker, Snail (D).

Supplementary Table 1: Primers used for site-directed mutagenesis of *Sur8*

Mutation	Orientation	Primer Sequence
T71A	Forward	5′-GCATTTTCAGTTGACAATGCGATCAAACGGCCAAACCCA -3′
	Reverse	5′-TGGGTTTGGCCGTTTGATCGCATTGTCAACTGAAAATGC-3′
T71E	Forward	5′-GCATTTTCAGTTGACAATGAGATCAAACGGCCAAACCCA-3′
	Reverse	5′-TGGGTTTGGCCGTTTGATCTCATTGTCAACTGAAAATGC-3′
S297A	Forward	5′-AGATATAACAGACTGGCAGCAATACCCAGATCA-3′
	Reverse	5′-TGATCTGGGTATTGCTGCCAGTCTGTTATATCT-3′
S297D	Forward	5′-CTGAGATATAACAGACTGGACGCAATACCCAGATCATTA-3′
	Reverse	5′-TAATGATCTGGGTATTGCGTCCAGTCTGTTATATCTCAG-3′
